# Self-Directed Recovery of Gu Syndrome: Reversal of Multisystem Dysfunction via Microbiome Restoration and Subconscious-Guided Protocols

**DOI:** 10.7759/cureus.94595

**Published:** 2025-10-14

**Authors:** Prabhjot K Chohan, Eric Brunhammer

**Affiliations:** 1 Complementary/Integrative Medicine, PlasmaMed Solutions Inc., Surrey, CAN; 2 Medical Affairs, Clear Energy Solutions, Wilmington, USA

**Keywords:** candida, diet modification, functional medicine, gu syndrome, leaky gut, microbiome, mold toxicity, sibo, subconscious healing, terrain theory

## Abstract

This case report documents a self-directed recovery from a complex, chronic multisystem condition consistent with Gu syndrome, involving *Candida* overgrowth, dysbiotic flora consistent with small intestinal bacterial overgrowth (SIBO), mold toxicity, intestinal hyperpermeability (leaky gut), and significant microbiome disruption. A 38-year-old male developed multiple symptoms after a trip to a developing country. His initial symptoms included excessive fatigue and weight gain, followed by multisystem involvement. Laboratory testing was positive for *Candida albicans*, dysbiotic flora consistent with SIBO, leaky gut, and mold toxicity. Management included dietary interventions, targeted supplementation, and intuitive subconscious guidance. The patient had a marked improvement in the clinical symptoms, physical and metabolic performance markers through a phased terrain-based recovery protocol and minimal pharmaceutical intervention. This case illustrates a novel integration of intuitive recovery and functional medicine with application for microbiome-centered therapeutic models.

## Introduction

Chronic multisystem illnesses involving fungal overgrowth, dysbiosis, and toxin-related inflammation are increasingly reported in both conventional and integrative medicine [1]. However, no single documented case has demonstrated laboratory-confirmed reversal of *Candida*, small intestinal bacterial overgrowth (SIBO), mold toxicity, and leaky gut with minimal pharmacological treatment.

The patient was diagnosed with Gu syndrome by a Chinese medicine practitioner after recovering from *Candida* and SIBO. Gu syndrome, according to Traditional Chinese Medicine (TCM), is a chronic inflammatory condition caused by pathogens, toxins, and parasites that resist conventional treatment. Dr. Heiner Fruehauf explains that Gu syndrome requires the presence of both digestive symptoms and neurological issues that are unexplained by conventional medicine or the usual TCM diagnostic patterns. There are two types of Gu syndrome: brain Gu, which involves brain fog, mood, and cognitive changes; and digestive Gu, characterized by altered bowel movements, bloating, and gas. Most cases also include chronic fatigue, muscle weakness, low-grade fever, and recurrent infections. Treatment usually involves detoxifying herbs, acupuncture, and holistic lifestyle changes [2].

This case supports terrain theory, as the patient restored his body's balance through nutrition and lifestyle changes. Terrain theory describes the body’s internal environment as a terrain that plays a crucial role in preventing disease and promoting healing. Based on Antoine Béchamp's view that a weakened or imbalanced terrain increases the risk of disease, this theory emphasizes lifestyle habits like proper nutrition, hydration, and rest for managing and preventing chronic illnesses [2,3]. Furthermore, the case highlights the potential influence of subconscious internal guidance on therapeutic choices, particularly since the patient was not under constant medical supervision. This subconscious guidance refers to the body's innate, intuitive intelligence or instinct that recognizes deeper emotional, psychological, and energetic patterns, beyond physical symptoms and lab results that influence a person’s health journey. It can be seen as a gut feeling or internal sense that subtly affects health decisions often outside of conscious awareness [2,4].

Therefore, this case report aims to demonstrate recovery from Gu syndrome using functional medicine protocols that focus on microbiome restoration or internal terrain, combined with subconscious guidance.

## Case presentation

The patient is a previously healthy 38-year-old Caucasian male who developed a cascade of symptoms within four months of a business trip to the Philippines in 2014.

Case progress timeline

2014-2018

The patient initially presented with unexplained weight gain and excessive fatigue. His starting weight was 180 pounds; he gained 30 pounds over six months, including a gain of 10 pounds in one day and 6 pounds on another day (see Appendices A-B). He experienced difficulty with activities of daily living. His physical performance declined, with his one-mile time increasing from five minutes to eight minutes and his 40-yard dash time rising from 4.45 seconds to 5.5 seconds. Mental symptoms included insomnia, dizziness, anxiety, low mood, and cognitive decline, characterized by trouble concentrating, confusion, and memory problems. Neurological symptoms included blurred vision, light sensitivity, and a sensation of pins and needles. Gastrointestinal symptoms included constipation and bloating, while genitourinary symptoms consisted of slow urination and nocturia.

The patient’s initial visit was to an endocrinologist in October 2014. Laboratory testing after the consultation showed a normal thyroid panel in January 2015. Laboratory testing included thyroid-stimulating hormone (TSH) and thyroid peroxidase (TPO), but thyroxine (T4) levels were not available in the records. However, elevated cortisol levels of 15.2 mcg/L (afternoon (PM) range: 4-9.9 mcg/L) and decreased total testosterone levels, from 832 ng/dL in 2011 to 405 ng/dL (193-950 ng/dL) in June 2015, were observed. The salivary cortisol indicated a mild elevation on a repeat test. Complete blood count (CBC), hemoglobin (Hb), hematocrit (Hct), TSH, and enteric pathogen bacterial stool culture with ova and parasites were all within normal limits, ruling out anemia and hypothyroidism. The endocrinologist recommended no repeat samples for ova and parasites; however, the possibility of an infection was ruled out with the negative stool culture. No specific treatment was recommended; the patient made dietary changes, excluding sugar and red meat, and started taking natural supplements such as digestive enzymes and betaine hydrochloride for digestion support, and a combination product with glutathione and essential minerals for detoxification.

The patient next had a consultation with a functional medicine physician in July 2015. His symptoms were persistent, although a physical exam was unremarkable. Blood tests done in August 2015 showed elevated low-density lipoprotein (LDL) particle at 1721 nmol/L (>1360 nmol/L), elevated high-sensitivity C-reactive protein (hsCRP) at 1.4 (<1), low coenzyme Q10 (CoQ) at 0.88 (<1.11) and sub optimal levels of vitamin D at 35 ng/mL (30-100 ng/mL). The stool analysis (GI effects test) identified growth of *Candida albicans* 2+, low bacterial diversity, and impaired fat absorption. He was prescribed nystatin 100,000 units oral suspension two times a day, which he consumed for a month. He started the Candida protocol, including the Candida diet - meat, eggs, vegetables, yogurt (MEVY) diet - and supplements in August 2015 and continued them until December 2016.

**Figure 1:**
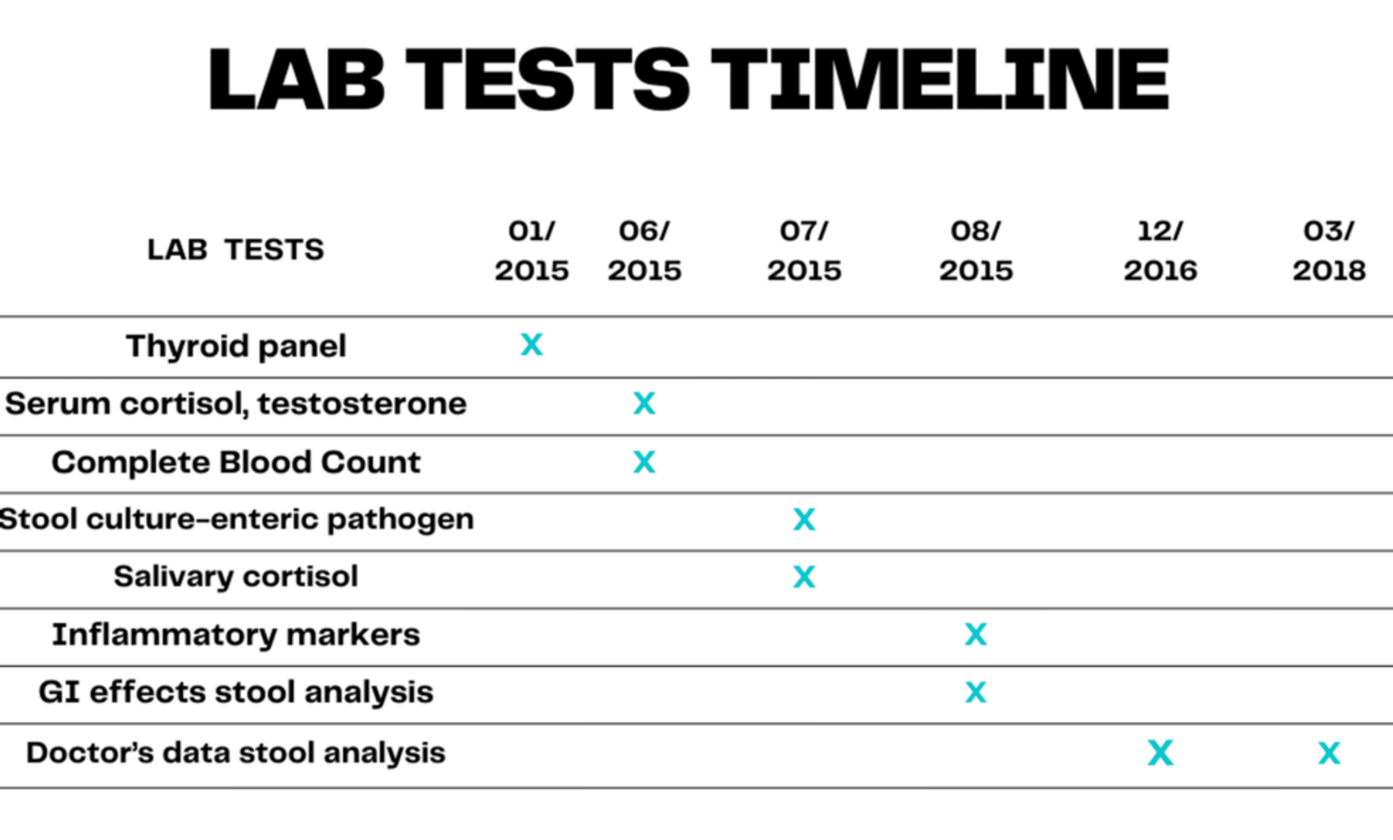
Timeline of laboratory tests done between 2015 and 2018.

Although the patient lost 20 pounds, experienced improved sleep quality and memory function, and noticed more regular bowel movements, he had not yet returned to his optimal physical performance and still experienced intermittent brain fog. In December 2016, he underwent a comprehensive stool analysis (as per the doctor’s data) based on a recommendation from a naturopathic doctor. The results confirmed the resolution of *C. albicans* but showed dysbiotic flora (*Citrobacter freundii* 3+), elevated fecal secretory immunoglobulin A (sIgA), and acidic stool. He began the SIBO protocol in December 2016 and continued until March 2018. Follow-up testing in March 2018, which included a repeat comprehensive stool analysis (doctor’s data), confirmed the resolution of dysbiotic flora consistent with SIBO (Figure 1, Table 1, and Appendices C-M).

**Table 1:** Laboratory tests conducted between 2015 and 2018, including results and reference ranges. Hematological tests showed normal complete blood count (CBC) values. Endocrine (hormonal) assessments revealed normal thyroid-stimulating hormone (TSH) levels, elevated cortisol levels, and decreased testosterone levels. The metabolic profile indicated increased low-density lipoprotein (LDL) particle concentration, while serological analysis showed mildly elevated high-sensitivity C-reactive protein (hsCRP). Functional medicine testing included comprehensive stool analyses performed before and after treatment with *Candida* and small intestinal bacterial overgrowth (SIBO) protocols. TPO: thyroid peroxidase antibody; MCV: mean corpuscular volume; MCH: mean corpuscular hemoglobin; MCHC: mean corpuscular hemoglobin concentration; MPV: mean platelet volume; RDW-CV: red cell distribution width-coefficient of variation; HDL-C: high-density lipoprotein cholesterol

Test date	Test name	Result	Normal range
01/15/2015	TPO	9.1 IU/ml	5.0-34.0 IU/ml
06/01/2015	TSH	1.73 moIU/ml	0.30-5.00 moIU/ml
	Cortisol	15.2 mcg/L	PM 4-9.9 mcg/L
	Testosterone	405.70 ng/dL	193.00-950.00 ng/dL
	CBC with differential		
	White Blood Cells	6.5 k/cu mm	3.6-10.2 k/cu mm
	Red Blood Cells	6.0 m/cu mm	4.4-6.0 m/cu mm
	Hemoglobin	15.0 gm/dL	13.2-18.0 gm/dL
	Hematocrit	45.90%	41.0-55.0%
	MCV	91 fL	82-99 fL
	MCH	30 pg	27-33 pg
	MCHC	33%	31-34%
	Platelets	230 k/cu mm	150-450 k/cu mm
	MPV	11.9 fL	9.8-12.7 fL
	RDW-CV	13%	11-15%
	Neutrophils	58%	37-72%
	Lymphocytes	28%	16-46%
	Monocytes	10%	4-14%
	Eosinophils	3%	0-9%
	Basophils	1%	0-2%
	Neutrophil count	3.8 k/cu mm	1.1-6.0 k/cu mm
	Lymphocyte count	1.8 k/cu mm	0.7-3.4 k/cu mm
	Monocyte count	0.7 k/cu mm	0.3-1.0 k/cu mm
	Eosinophil Count	0.2 k/cu mm	0.0-0.6 k/cu mm
	Basophil Count	0.0 k/cu mm	0.0-0.1 k/cu mm
07/09/2015	Stool culture-Enteric pathogen, ova and parasites	Negative for bacteria, ova and parasites	No growth
07/22/2015	Salivary cortisol-Genova diagnostics		
	7 am-9 am	1.12 mcg/dL	0.27-1.18 mcg/dL
	11 am-1 pm	0.38 mcg/dL	0.10-0.41 mcg/dL
	3 pm-5 pm	0.18 mcg/dL	0.05-0.27 mcg/dL
	10 pm-12 am	0.22 mcg/dL	0.03-0.14 mcg/dL
08/01/2015	Low-density lipoprotein (LDL) particle	1721 nmol/L	>1360 nmol/L (high risk)
	Apo B	83 mg/dL	>80 mg/dL (high risk)
	Total cholesterol	158 mg/dL	<200 mg/dL (optimal)
	LDL-C Direct	81 mg/dL	<100 mg/dL (optimal)
	HDL-C	41 mg/dL	>40 mg/dL (optimal)
	High-sensitivity C-reactive protein (hsCRP)	1.4 mg/L	1.0-2.9 mg/L (intermediate risk)
	Coenzyme Q10 (CoQ)	0.88	<1.11
	25-Hydroxy vitamin D	35 ng/mL	30-100 ng/mL
08/04/2015	Stool analysis -GI effects test		
	Microbiome	Candida albicans 2+ Lactobacillus spp-No growth	Candida albicans-No growth Lactobacillus spp-1+-4+
	Fecal fat total	>51 mg/g	3.2-38.6 mg/g
12/05/2016	Comprehensive stool analysis-Doctor’s Data		
	Microbiome	No yeast isolated Dysbiotic flora detected	No growth of yeast Normal flora
	Stool IgA	489 mg/dL	51-204 mg/dL
	Stool pH	5.9	6-7.8
03/03/2018	Comprehensive stool analysis-Doctor’s Data		
	Microbiome	No yeast isolated Normal flora	No growth of yeast

The patient had no allergies; however, he experienced mild to moderate adverse reactions. These included episodes of increased brain fog, fatigue, and weight gain, mainly while following the Candida protocol. He monitored his weight using a weighing scale and kept a journal of his experiences. The supplement choices were based on research, with his subjective response guided by subconscious intuition. Although he adhered to the dietary protocols, his food intake was adjusted according to his satiety levels.

2018-2022

The patient had a general check-up with a Chinese medicine doctor and was diagnosed with residual Gu syndrome in April 2020. The chronic digestive issues, such as Candida and SIBO, along with cognitive symptoms and recent infection after foreign travel, indicated Gu syndrome. The patient still experienced slow urination, consistent with fluid metabolism problems, including spleen damp cold and bladder damp cold. Some emotional volatility might be caused by excess heat in the heart and liver, reflecting a history of liver/heart fire. A physical exam was unremarkable except for active discomfort at liver and stomach mu points. The tongue showed a thick white coat in the large intestine area, slight cracks and depression in the center, and excess heat in the liver and heart, with some swelling in the liver regions. The pulse was muffled, wiry on the left side, and slippery on the right side (Appendices N-P). The doctor recommended a Chinese formula, but the patient declined and continued his research. He followed the Mediterranean diet from March 2018 to July 2020.

In July 2020, the patient self-ordered an intestinal antigenic permeability screen (Cyrex array), which showed increased levels of occludin/zonulin IgA, lipopolysaccharide (LPS) IgG, and IgA, indicating a leaky gut. Subsequently, he began the leaky gut protocol in July 2020 and continued it until April 2022. The intestinal antigenic permeability screen was repeated in April 2022 and showed normal levels of occludin/zonulin IgA, LPS IgG, and elevated IgA, suggesting improved gut integrity. The patient experienced die-off symptoms such as brain fog and headache during this phase. Additionally, the patient had confirmatory SIBO breath tests in March 2021 and April 2022, both within normal limits.

**Figure 2:**
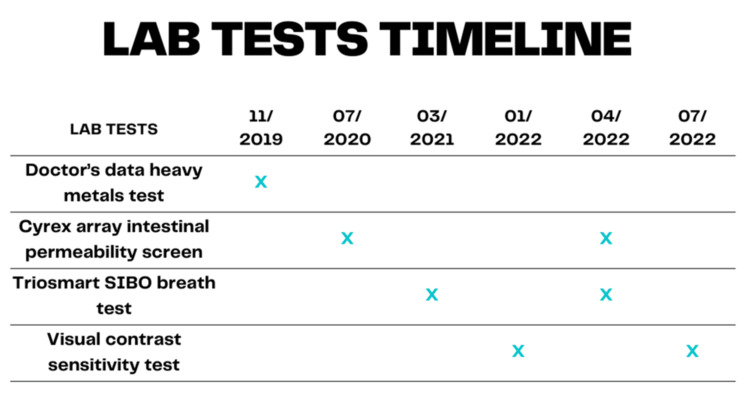
Timeline of the laboratory tests done between 2019 and 2022. SIBO: small intestinal bacterial overgrowth

The patient noted a significant improvement in his fatigue, body composition, neurological, and gastrointestinal symptoms. However, urinary symptoms had a gradual progression, and he still had slow urination. Subsequently, mold exposure was detected in January 2022 through the Visual Contrast Sensitivity (VCS) test, which identifies common species of mold, including *Stachybotrys*, *Aspergillus*, and *Penicillium*. A VCS test can produce false positives due to heavy metal toxicity and nutritional deficiencies. A negative heavy metals test was performed in November 2019, ruling out heavy metal toxicity. The patient started a mold detoxification protocol in January 2022 and continued it until July 2022. This led to normalized test results by July 2022, supporting mold-related involvement. However, the patient experienced weight gain during this period as an adverse reaction (Figure 2, Table 2, and Appendices C-M).

**Table 2:** Laboratory tests conducted between 2019 and 2022, including results and reference ranges. Functional medicine testing included a negative heavy metals screen, a negative small intestinal bacterial overgrowth (SIBO) breath test, and pre- and post-treatment results for the intestinal antigenic permeability screen and visual contrast sensitivity test.

Test Date	Test name	Result	Normal range
11/22/2019	Heavy metals test- Doctor’s data	Negative	Negative
07/10/2020	Intestinal antigenic permeability screen- Cyrex array		
	Occludin/Zonulin IgA	2.49	0.1-1.6
	Lipopolysaccharide (LPS) IgG	2.68	0.0-2.6
	Lipopolysaccharide (LPS) IgA	>2.80	0.0-1.8
03/28/2021	SIBO breath- Triosmart test		
	Hydrogen	7.22 ppm	<26.79 ppm
	Methane	3.01 ppm	<10.00 ppm
	Hydrogen sulfide	2.30 ppm	<5.00 ppm
01/21/2022	Visual contrast sensitivity test		
	Total score	38/90 (42%) Positive	100
	Biotoxin score	13/36 (36%) Positive	100
04/20/2022	Intestinal antigenic permeability screen- Cyrex array		
	Occludin/Zonulin IgA	0.61	0.1-1.6
	Lipopolysaccharide (LPS) IgG	1.94	0.0-2.6
	Lipopolysaccharide (LPS) IgA	>2.80	0.0-1.8
04/24/2022	SIBO breath-Triosmart test		
	Hydrogen	4.78 ppm	<24.96 ppm
	Methane	0.90 ppm	<10.00 ppm
	Hydrogen sulfide	2.16 ppm	<3.00 ppm
07/05/2022	Visual contrast sensitivity test		
	Total score	90/90 (100%) Negative	100
	Biotoxin score	36/36 (100%) Negative	100

The patient followed intervention phases, which included pathogen elimination, detoxification, gut repair, and regeneration managed with dietary modification and supplements (Table 3). He managed the extended periods of healing using self-monitoring strategies such as keeping a detailed journal of body measurements (chest, arms, thighs, calves, waist), daily body weight, and notes on mental and emotional well-being. Each entry included reflections on which protocols were effective, helping him track trends over time and maintain adherence. His weight dropped to 176 pounds, along with an increase in muscle mass and improved recovery of his torn Achilles tendon. The patient’s other symptoms, including fatigue, neurological, gastrointestinal, and urinary, also showed complete resolution.

**Table 3:** Intervention phases across different stages of healing. FODMAPs: fermentable oligosaccharides, disaccharides, monosaccharides, and polyols

Phase	Category/Focus
Phase 1	Pathogen Elimination
Botanicals	Oregano oil, berberine, garlic, caprylic acid
Diet	Meats, eggs, vegetables, yogurt (MEVY diet for *Candida*), restrictive diets
Phase 2	Detoxification
Binders	Activated charcoal, glutathione, chlorella, N-acetyl cysteine
Phase 3	Gut Repair
Mucosal support	Slippery elm, marshmallow root, glycine, gelatin
Regeneration	L-glutamine
Phase 4	Microbiome Rebuilding
Bifidobacterium bifidum	Inulin, human milk oligosaccharides (HMO), agave
Lactobacillus acidophilus	Acidic foods, FODMAPs
Escherichia coli	Spirulina, carbon sources, acetate precursors

## Discussion

The patient worked as an energy trader and frequently traveled nationwide and internationally for business. He was a health enthusiast, exercised five days a week, and followed a very healthy diet. He was a non-smoker, non-alcoholic, and avoided recreational drugs. His initial symptoms were triggered after staying in a developing country for two months. Stool testing revealed yeast overgrowth and dysbiotic flora, indicating SIBO.

While the factors typically linked to *Candida* growth, such as an unhealthy diet high in fat and sugar, low in fiber, a sedentary lifestyle, prolonged psychological stress, antibiotic use, excessive smoking, and alcohol consumption [5], were not present in this case, the patient reported a significant improvement in symptoms and better test results for *Candida* overgrowth after making dietary changes. The patient eliminated sugar as part of the protocol for the Candida and MEVY diets. However, sugar and additional fiber in the form of low fermentable oligosaccharides, disaccharides, monosaccharides, and polyols (FODMAPs) [6] were included in the SIBO diet. The next step was to address the microbes. Changes in the gut microbiome [5], including alterations in the *Firmicutes*-to-*Bacteroidetes* ratio (typically 60-80% *Firmicutes* and 20-40% *Bacteroidetes*), were considered in the patient’s treatment plan [7]. Botanical antimicrobials, such as garlic, thyme, coconut oil, olive oil, berberine, and oregano, have been shown to reduce the growth of fungi and bacteria, resulting in symptom relief and improved clinical outcomes [5].

Despite normal stool test results, leaky gut persisted, shown by an intestinal antigenic permeability screen. Research suggests that gut dysbiosis causes the unregulated secretion of zonulin, a protein that disrupts the gut barrier and alters endotoxins and antigens. This initiates an immune response with the release of cytokines [8] and stimulation of mucosal release of IgA [7]. The patient began the leaky gut protocol with restrictive diets to address the ongoing gut lining permeability and target specific microbes. Evidence supports the efficacy of restrictive diets such as a low histamine diet [9], a low sulfur diet [10], a diet with low H2S producing bacteria [11] and a plant-based diet in significantly altering the composition of the gut microbiome, increasing beneficial commensals in the gut, such as *Bifidobacterium bifidum* and *Lactobacillus acidophilus*, while reducing pathogenic *Bacteroides fragilis* and *Clostridium perfringens* [5]. Similarly, bacteriophage communities, termed viromes, can be influenced by factors such as age, gender, diet, lifestyle, disease, and medication, significantly impacting the bacteriome [12]. The herbs and supplements, such as slippery elm, marshmallow, glycine, and glutamine, were included to aid in the repair of the epithelial lining [13]. Prebiotics, such as inulin, provide fermentable fibers that decrease the number of *Firmicutes* while probiotics directly introduce beneficial commensal bacteria [12]. Furthermore, carbon sources such as glucose and acetate serve as media for the growth of favorable strains of *Escherichia coli* [14]. The persistence of mold on laboratory tests could be explained by evidence that illustrates mold and mycotoxins as potential modulators of the immune system. Mycotoxins trigger the inflammatory responses and prolong the existing immunosuppressed states. Supplements such as glutathione and N-acetyl cysteine, which facilitate detoxification and possess antioxidant properties, effectively combat mold [15].

The patient’s treatment phases, including pathogen elimination, detoxification, gut repair, and regeneration, aligned with the traditional 5R protocol in functional medicine, which consists of Remove, Replace, Reinoculate, Repair, and Rebalance [16]. Both laboratory confirmation of resolution and subjective assessment of symptoms determined the phase transition. A normalized test result alone was not enough; intuition about overall health and symptoms progression guided the decision to advance to the next stage. The adverse reactions included both temporary exacerbations similar to the "Jarisch Herxheimer reaction" and non-Herxheimer reactions. The former are common in infection treatments when symptoms worsen as pathogens are killed, releasing toxins [17], while the latter can be attributed to the supplements. Since the patient was not under constant medical supervision, he relied on his intuition to distinguish adverse reactions and used terrain theory to choose appropriate supplements [3]. Weight was tracked using a digital scale as an objective marker of inflammation. The patient noted rapid weight gain as a sign of inflammation and weight loss as a sign of decreased inflammation. For example, during mold management, methylsulfonylmethane (MSM) was used, resulting in an initial four-pound weight loss. MSM contains sulfur that removes mold; however, its sulfur metabolism pathways were downregulated, leading to weight gain after continuing MSM for six months. He then discontinued MSM, a decision supported by ongoing weight tracking, his bodily responses, and intuitive judgment. Evidence shows that improvements in serum markers of inflammation are associated with reduced body weight [18].

The subconscious-driven approach may be especially relevant for addressing the deep mental, emotional symptoms, and physical patterns linked to Gu syndrome [2]. This method aligns with traditional Chinese and indigenous healing paradigms, where the practitioner begins by listening inward and engaging in self-treatment. However, subconscious guidance is highly subjective, relying on an individual’s thoughts and expressions. Furthermore, inconsistencies in dosing various supplements can limit their reliability for recovery [19]. Another issue with relying on subconscious guidance is choosing the correct laboratory tests. For example, *Candida* overgrowth and SIBO highlight terms from functional medicine and were not verified by gold-standard diagnostic methods. For clarity, these can also be called "intestinal fungal overgrowth" and "dysbiotic flora consistent with SIBO." Similarly, a VCS test is a screening tool, not a definitive diagnostic test for mold. No urine mycotoxin screens or antibody panels were performed to confirm mold presence. The mold diagnosis remains presumptive, based on functional testing rather than gold-standard laboratory confirmation.

The case met the key diagnostic criteria for Gu syndrome, involving multisystem issues such as extensive neurological and digestive symptoms, with detection of fungal and bacterial overgrowth without other risk factors. The patient’s presentation was not explained by conventional medicine or the usual TCM patterns [2,20]. The Gu syndrome management includes dietary interventions, lifestyle changes, acupuncture, and herbs, and minimal pharmaceutical interventions. This correlates with Fruehauf's perspective on the Gu syndrome treatment, which avoids using non-tonic, strong medications that can be difficult to digest and give only temporary relief [2]. The patient followed different protocols sequentially: Candida for about two years, SIBO for another two years, mold for six months, followed by gut lining repair for a year, and regrowth of bacteria for two to three years. Although extended treatment phases are prone to plateaus, the patient was able to evaluate his improvement retrospectively by journaling his emotional state, coupled with objective tracking of his anthropometric measurements and fitness performance metrics. The process of systematically tracking and visually reviewing these changes appeared to reinforce motivation, creating a positive feedback loop that contributed to sustained adherence and facilitated self-directed recovery [2].

## Conclusions

This case is unique because it shows lab-confirmed normalization of *Candida*, dysbiotic flora consistent with SIBO, mold, leaky gut, and systemic Gu syndrome with dietary interventions, supplementation, and minimal use of pharmaceuticals. It provides a foundation for integrating microbiome-focused protocols in functional medicine, terrain theory, and subconscious guidance. A subconscious-driven approach could be relevant in addressing the deeply rooted mental and physical patterns associated with Gu syndrome. This is a single case, and the results would only be generalizable with further research studies. Future research should evaluate subconscious-driven decision models as a therapeutic strategy for complex chronic inflammatory conditions such as fibromyalgia, chronic fatigue syndrome, and autoimmune diseases that are considered persistent or idiopathic.

## Figures and Tables

**Figure 3:**
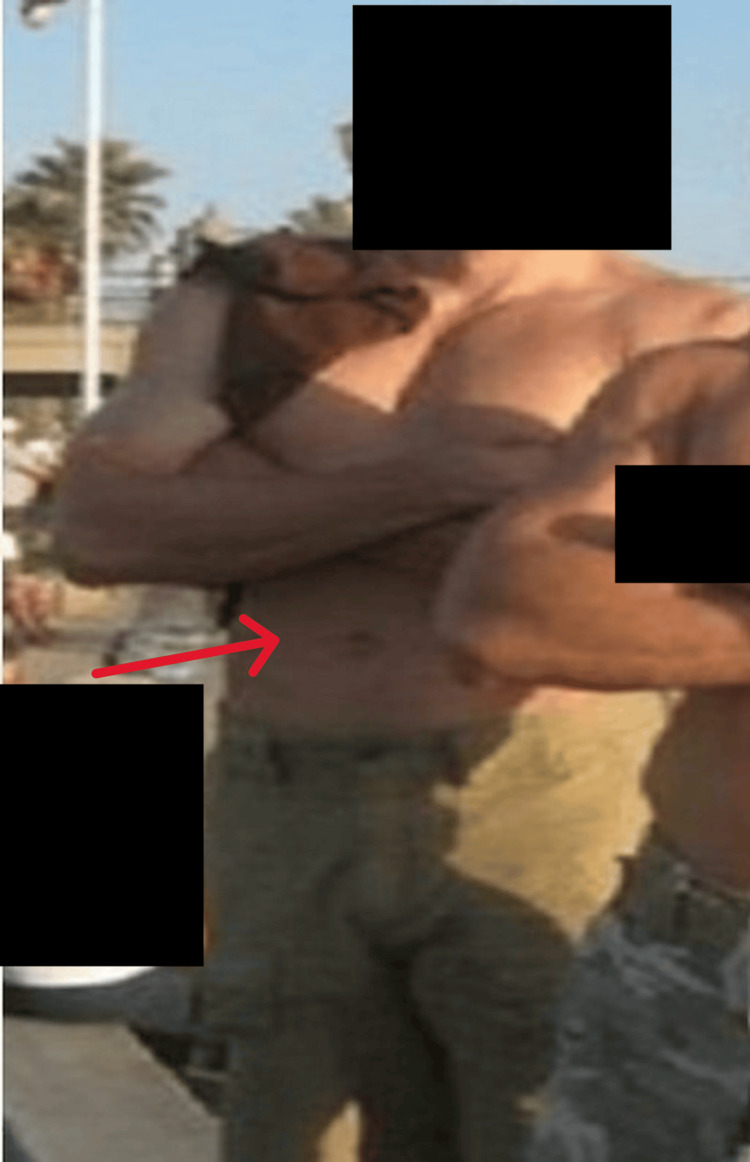
Patient’s body composition prior to traveling to the Philippines in September 2014. His initial weight was 180 pounds.

**Figure 4:**
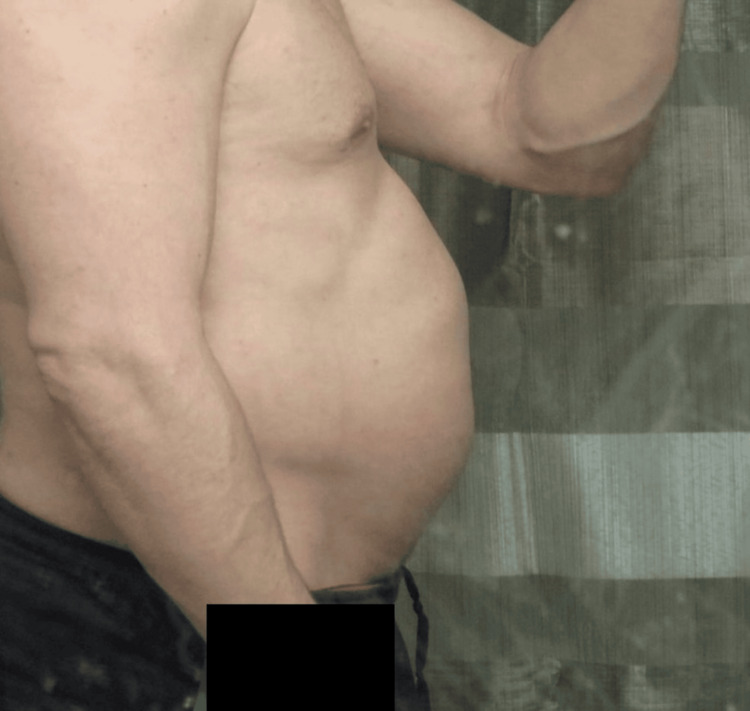
Patient’s body structure after he returned from the Philippines in November 2014. He gained 30 pounds over six months.

**Figure 5:**
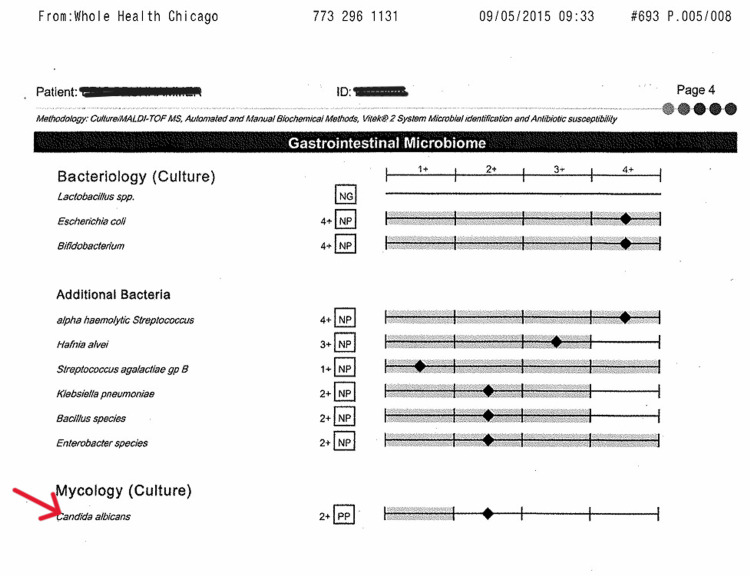
Stool analysis - gastrointestinal effects test conducted before treatment with the Candida protocol (sample collected on 08/04/2015). The red arrow indicates a 2+ positive result for Candida albicans.

**Figure 6:**
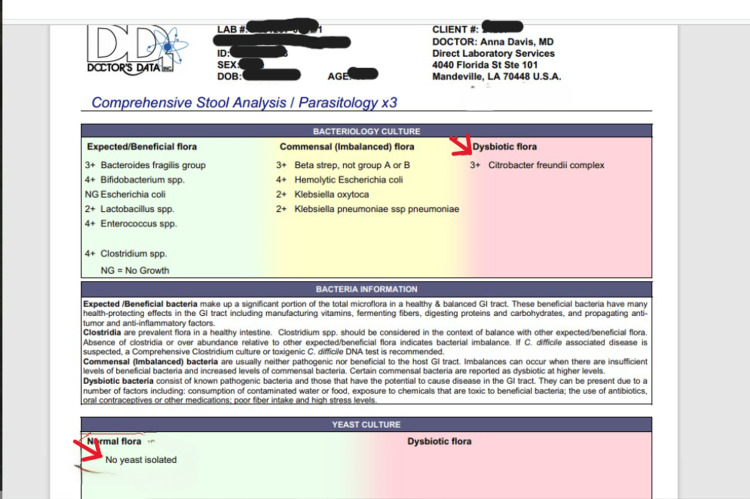
Comprehensive stool analysis (Doctor’s Data) following treatment with the Candida protocol (sample collected on 12/05/2016). The red arrows indicate no yeast growth (Candida albicans) and 3+ dysbiotic flora (Citrobacter freundii), consistent with small intestinal bacterial overgrowth (SIBO).

**Figure 7:**
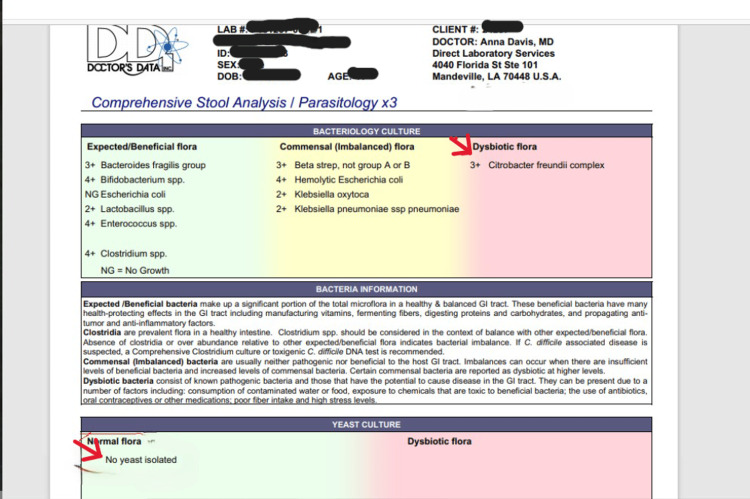
Comprehensive stool analysis (Doctor’s Data) prior to treatment with the small intestinal bacterial overgrowth (SIBO) protocol (sample collected on 12/05/2016). The red arrows indicate 3+ dysbiotic flora (Citrobacter freundii), consistent with SIBO, and no yeast growth (Candida albicans).

**Figure 8:**
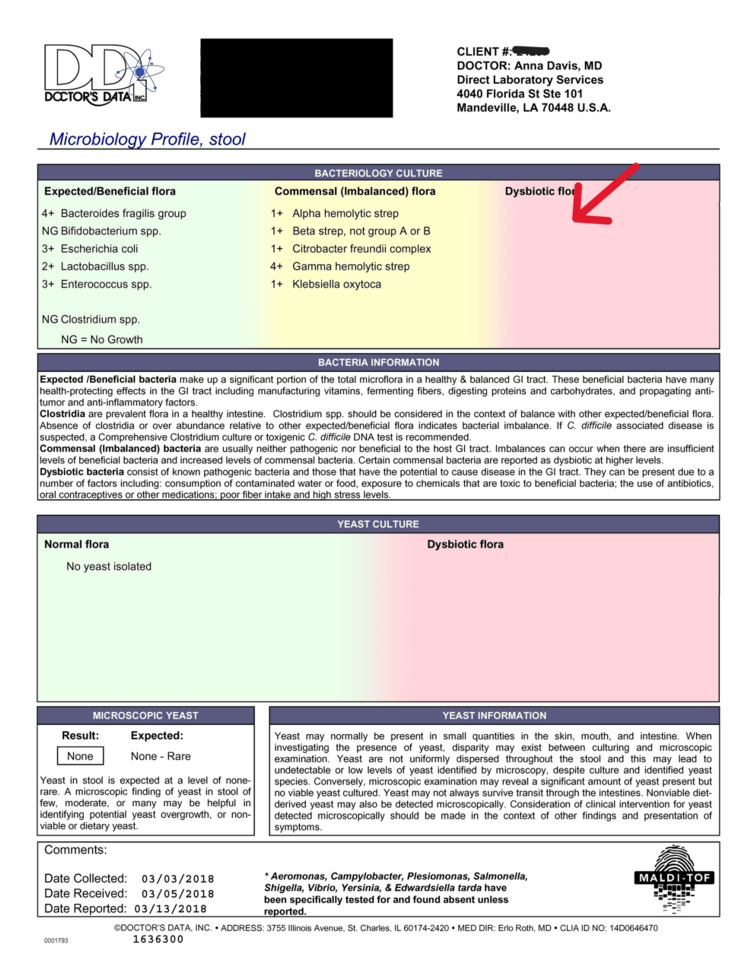
Comprehensive stool analysis (Doctor’s Data) following treatment with the small intestinal bacterial overgrowth (SIBO) protocol (sample collected on 03/03/2018). The red arrow indicates a negative result for dysbiotic flora, consistent with resolution of SIBO.

**Figure 9:**
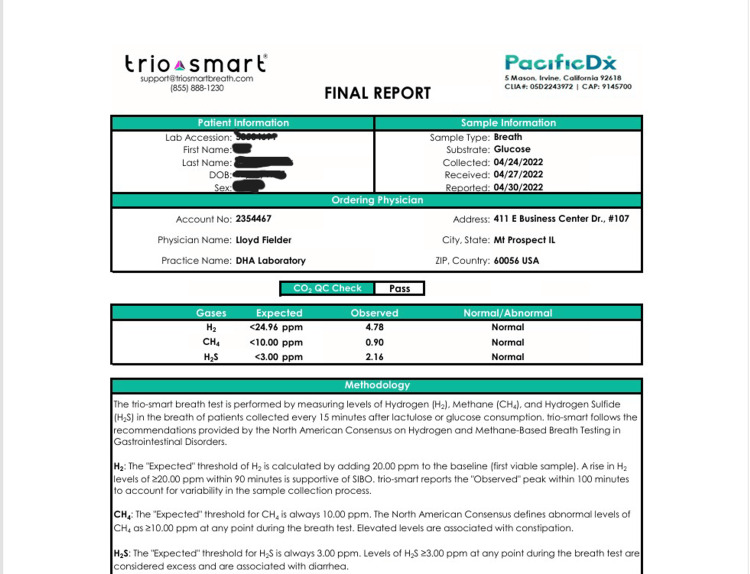
TrioSmart breath test following treatment with the small intestinal bacterial overgrowth (SIBO) protocol (sample collected on 04/24/2022). Results show normal levels of hydrogen, methane, and hydrogen sulfide, indicating a negative result for dysbiotic flora consistent with resolution of SIBO.

**Figure 10:**
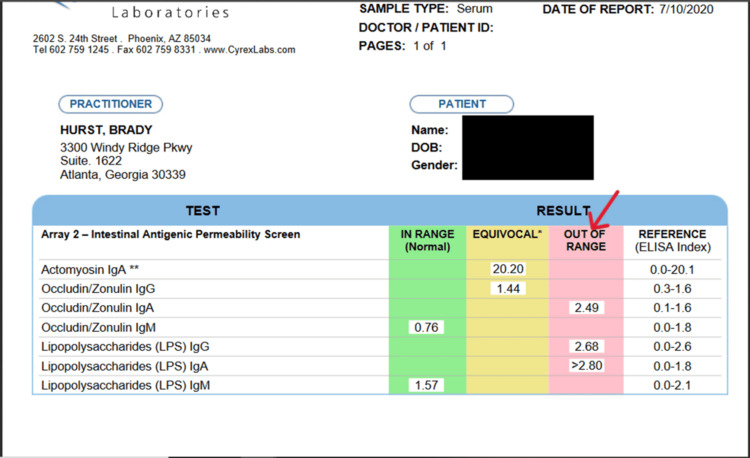
Cyrex Array intestinal antigenic permeability screen prior to treatment with the leaky gut protocol (report dated 07/10/2020). The red arrow indicates elevated levels of occludin/zonulin IgA (2.49), lipopolysaccharide IgG (2.68), and IgA (>2.80), consistent with intestinal permeability (“leaky gut”).

**Figure 11:**
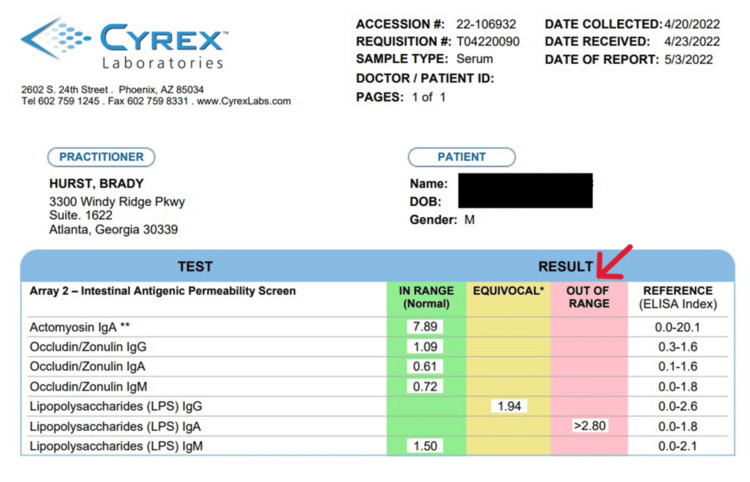
Cyrex Array intestinal antigenic permeability screen following treatment with the leaky gut protocol (sample collected on 04/20/2022). Results show normal occludin/zonulin IgA levels, equivocal lipopolysaccharide IgG levels, and elevated lipopolysaccharide IgA (>2.80, indicated by the red arrow). These findings suggest improvement in intestinal permeability (“leaky gut”).

**Figure 12:**
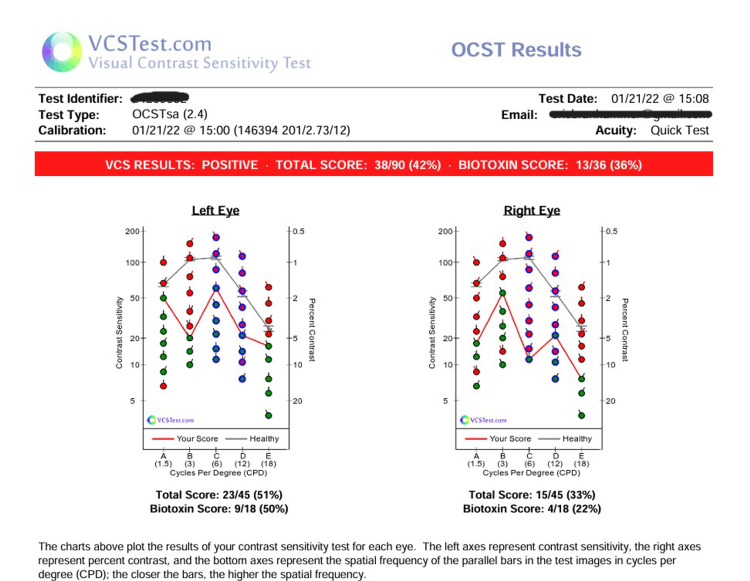
Visual contrast sensitivity test prior to treatment with the mold protocol (sample collected on 01/21/2022). Results show a total score of 38/90 (42%) and a biotoxin score of 13/36 (38%), indicating possible mold exposure.

**Figure 13:**
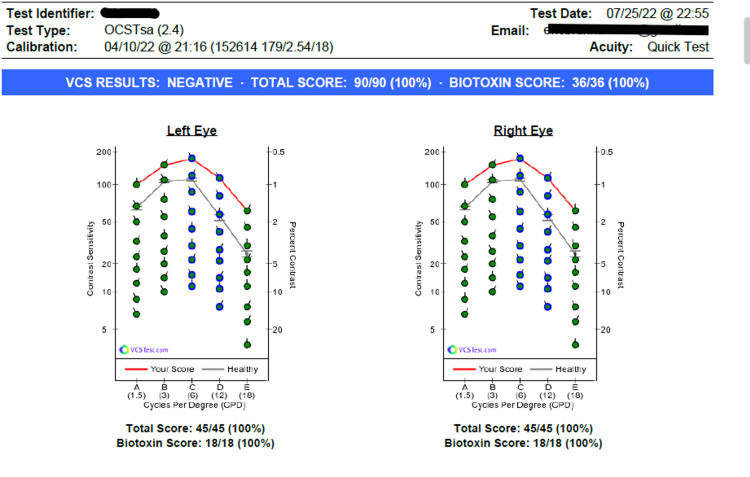
Visual contrast sensitivity test following treatment with the mold protocol (sample collected on 07/25/2022). Results show normal total and biotoxin scores, indicating possible resolution of mold exposure.

**Figure 14:**
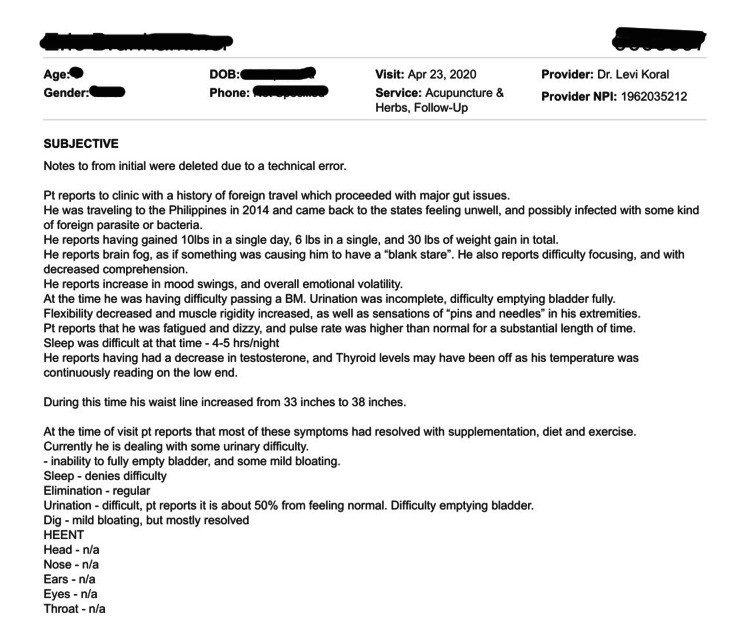
Subjective section of the chart note from the patient’s visit to the Chinese medicine doctor (04/23/2020). The image displays the history of present illness and review of systems.

**Figure 15:**
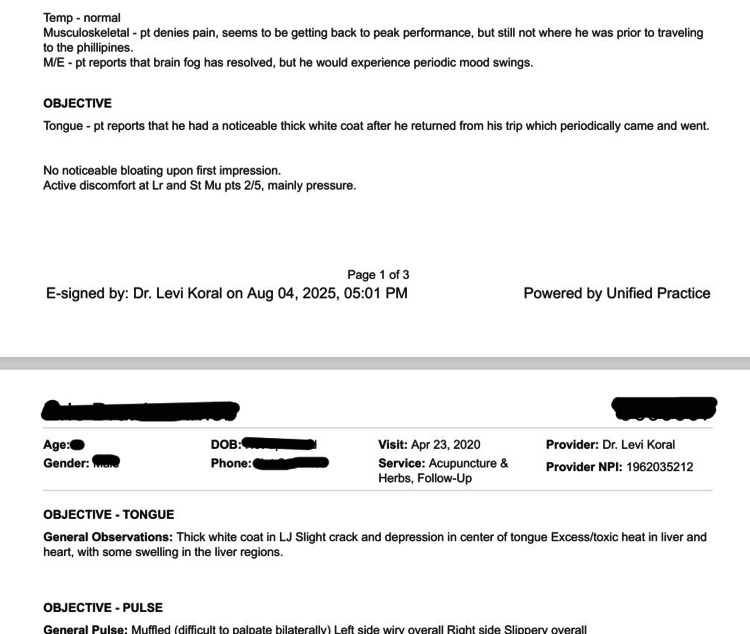
Objective section of the chart note from the patient’s visit to the Chinese medicine doctor (04/23/2020). The image shows no noticeable abdominal bloating on physical examination, except for tenderness at the liver and stomach mu points. The tongue exhibits a thick white coating in the large intestine area, slight central cracks and depression, and signs of excess heat in the liver and heart, with mild swelling in the liver regions. The pulse is muffled and wiry on the left side, and slippery on the right side.

**Figure 16:**
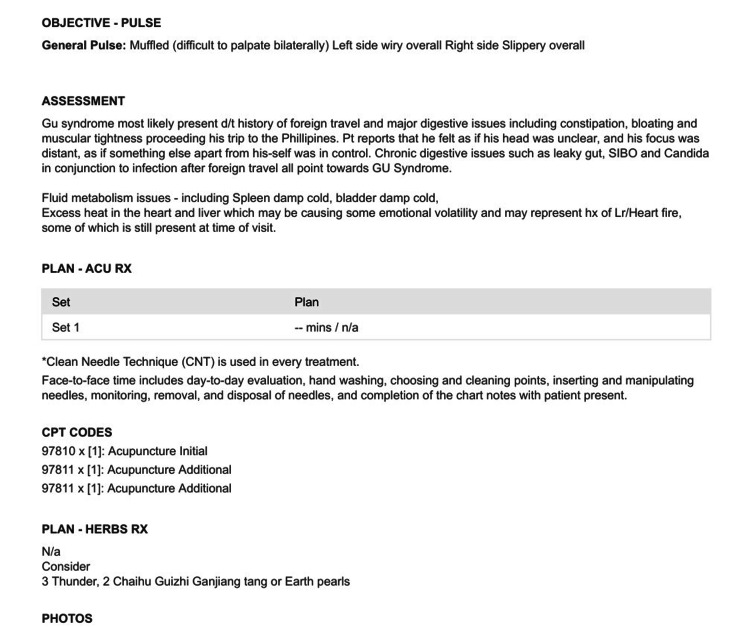
Assessment and plan sections of the chart note from the patient’s visit to the Chinese medicine doctor (04/23/2020). The image indicates a diagnosis of Gu syndrome and the doctor’s recommendation of a Chinese herbal formula.

## Appendices

Appendix A

The Patient's Pictures Before and After the Visit to the Philippines

Appendix B

Appendix C

Timeline of Patient’s Symptoms, Adverse Reactions, Laboratory Tests, and a Description of Protocols, Including Diet and Supplements

**Table 4:** Timeline of patient’s symptoms, adverse reactions, laboratory tests, and a description of protocols including diet and supplements from 2014 to 2018. TSH: thyroid-stimulating hormone; TPO: thyroid peroxidase; SIBO: small intestinal bacterial overgrowth; CBC: complete blood count; MEVY: meat, eggs, vegetables, and yogurt; HCl: hydrochloric acid

Timeline	Case Progression, Recovery, Protocols
January 2015	Thyroid panel - TSH, TPO normal
Symptoms	Constitutional: Approximately 10 pounds of weight gain in a single day, 6 pounds of weight gain on another day, about 30 pounds of total weight gain, fatigue, an increase in 1-mile run time from 5 minutes to 8 minutes, and a 40-yard dash time increased from 4.45 seconds to 5.5 seconds. (1 mile time is the time taken to run a mile, 40-yard dash time is the time taken to run a yard.) Musculoskeletal: Decreased flexibility, increased stiffness, and inability to do a split. Mental symptoms: Insomnia, dizziness, anxiety, low mood, and cognitive decline, characterized by difficulty concentrating, confusion, and memory issues. Neurological symptoms: Blurred vision, light sensitivity, blank stare, and a sensation of pins and needles. Gastrointestinal symptoms: Constipation and bloating. Genitourinary symptoms: Slow urination and nocturia. Temperature: 95°F, high pulse rate.
June 2015	Serum cortisol elevated, testosterone decreased, CBC and TSH normal
July 2015	Enteric pathogen stool culture, ova and parasites normal, salivary cortisol elevated
August 2015	Markers of inflammation elevated, *Candida albicans* positive - GI effects test
Candida protocol used from August 2015 to December 2016, and adverse reactions	MEVY diet: included meat, eggs, vegetables, and yogurt. This diet consisted of chicken breast, filet, eggs, olive oil, vegetables consisting of carrots, lettuce, green peppers, cucumber, and no sugar Greek-style yogurt. Candida diet: included a low sugar, anti-inflammatory diet consisting of chicken, steak, varied vegetables, complex carbohydrates including brown rice and quinoa, no added sugar, minimal to no natural sugar. Dietary intake: six meals per day. Each meal contained 20% protein, 20% fat, and 60% carbohydrates. Supplements used: Oregano 150-300 mg/day 1 cap 1-2x/day, berberine 500 mg 1 cap 2-3x/day, garlic 600-1200 mg/day 1 cap 2-3x/day, caprylic acid 500-1,000 mg/day 1 cap 2-3x/day, biofilm buster 2 caps/day, digestive enzymes with HCl - four caps taken with protein meals, multi-strain probiotics 20-30 billion CFUs/day, trace minerals - 2 capsule/day, nystatin 100,000 units oral suspension 2x/day for a month. Antifungals - oregano, berberine, garlic, caprylic acid - were rotated from three days to one week. Brain fog, die off, flu-like symptoms.
December 2016	*C. albicans* resolved, dysbiotic flora detected - Doctor’s data, stool analysis
Symptoms: Progression after *Candida* recovery	Constitutional: Fatigue improved by 70%. Body composition improved by 50%. The patient’s weight decreased from 210 pounds to 190 pounds. The patient’s waist was 34 inches, but in his previous peak condition, it was 31 inches at 180 pounds. Run times improved by 50%; 40-yard dash time was not timed. The 1-mile time was around 6:15 minutes. Musculoskeletal: Improved flexibility, decreased stiffness 70%, the patient could do a split. Mental symptoms: Insomnia improved 70%, dizziness resolved, mood swings 75% improved, and difficulty thinking and understanding 90% improved. Neurological symptoms: Blurred vision resolved, blank stare resolved, sensation of pins and needles 85% improved, light sensitivity 60% improved. Gastrointestinal symptoms: Constipation resolved, consistency of stool hard to semi-solid, bloating resolved. Genitourinary symptoms: Urination improved 10% Vitals: High pulse rate 90% resolved, low body temperature 65% resolved. Lab tests: Prediabetes: unknown, not retested. Testosterone: unknown, not retested.
SIBO protocol used from December 2016 to March 2018, and adverse reactions	Diet: Lean protein, including turkey, chicken breast, filet, eggs, carbohydrates including white and brown rice, quinoa, sweet potatoes, sourdough bread, nuts and seeds, olive oil, varied vegetables with higher amounts of fiber and natural sugar from bananas, figs, cherries, mangos, watermelon, and coconut milk. Dietary intake: About 6 meals per day. Each meal contained 20% protein, 20% fat, and 60% carbohydrates. Supplements: Pau D'arco - 500-1,000 mg 1 cap 1-2x/day, Olive leaf - 500-1,000 mg/day 1 cap 1-2x/day, Vitamin C - 500-2,000 mg/day, liquid zinc - 15-30 mg elemental zinc/day, B-12 - 1,000-2,000 mcg/day, ginger - 500-2,000 mg/day, oregano - 100-200 mg/day, berberine - 500 mg 1 cap 2-3x/day, Neem - 500-1,000 mg/day 1 cap 1-2x/day, Allicin 180-600 mg/day 1 cap 2-3x/day. No adverse reactions to supplements or diet.
March 2018	*C. albicans* negative, normal flora - Doctor’s data, stool analysis
Symptoms: Progression after SIBO recovery	Constitutional: Fatigue improved by 85%. Body composition improved by 65%. The patient’s weight decreased from 210 pounds to 190 pounds. The patient’s waist was 33 inches; in previous peak condition, it was 31 inches at 180 pounds. Fatigue improved by 50%, run times improved by 65%, 40-yard dash time was not timed. The 1-mile time was around 05:55 minutes. Musculoskeletal: Improved flexibility, stiffness 85% improved, split possible. Mental symptoms: Insomnia improved 85%, mood swings 80% improved, and difficulty thinking and understanding 95% improved. Neurological symptoms: Sensation of pins and needles 95% improved, light sensitivity 85% improved. Gastrointestinal symptoms: Constipation resolved, and the quality of stool improved; bloating resolved. Genitourinary symptoms: Urination improved 50%. Vitals: High pulse rate resolved, low body temperature: 85% resolved.

Appendix D

**Table 5:** Timeline of patient’s symptoms, adverse reactions, laboratory tests and a description of protocols including diet and supplements from 2018 to 2022.

Timeline	Case Progression, Recovery, Protocols
April 2020	General check-up with a Chinese medicine doctor. Diagnosis of Gu syndrome given
July 2020	Leaky gut detected: Intestinal antigenic permeability screen: Cyrex array
Leaky gut protocol used from July 2020 to April 2022, and adverse reactions	Leaky gut was a complex and multifaceted protocol. Multiple bacterial strains and viruses were treated. Multiple and varied diets and supplements were used. Die off symptoms such as brain fog, headache.
March 2021	SIBO breath test negative - Triosmart test
April 2022	SIBO breath test negative - Triosmart test
January 2022	Mold detected - Visual contrast sensitivity test
Mold protocol used from January 2022 to July 2022, and adverse reactions	Mold diet: Chicken, almonds, olive oil, lettuce, broccoli, carrots, cucumber, peppers, tomatoes, potatoes, brown and white rice, whole wheat bread, almond milk, Pellegrino, spring water. Dietary intake: About 6 meals per day. Each meal contained 20% protein, 20% fat and 60% carbohydrates. Supplements: Glutathione 250-500mg 1 cap/Day, N-acetyl cysteine 600-1,800 mg/day, 1 cap 2-3x/day quercetin 500-1,000 mg/day 1 cap 2x/day, MSM 500-3,000 mg/day, 1 cap 2-3x/day. Weight gain
April 2022	Leaky gut resolving - Intestinal antigenic permeability screen - Cyrex array
Symptoms: Progression after leaky gut recovery	Constitutional: Severe fatigue resolved. Body composition improved by 65%. The patient’s weight decreased from 190 pounds to 180 pounds in 4 days. Run times improved by 85%, 40-yard dash time was not timed. The 1-mile time was around 05:35 minutes. Musculoskeletal: Improved flexibility, stiff muscles resolved, the patient could do a split for 20 minutes. Mental symptoms: Insomnia improved 90%, mood swings 80% improved, difficulty thinking and understanding resolved. Neurological symptoms: Sensation of pins and needles resolved, light sensitivity resolved, mood swings 90% resolved. Gastrointestinal symptoms: Constipation resolved and quality of stool improved, bloating resolved. Genitourinary symptoms: Urination improved 65%. Vitals: High pulse rate resolved, low body temperature resolved.
July 2022	Mold resolved - Visual contrast sensitivity test
Symptoms: Progression after mold recovery	Constitutional: Severe fatigue resolved. Body composition was improving. The patient’s weight decreased from 180 pounds to 176 pounds. Run times improved by 90%, 40-yard dash time was timed at 4:38 seconds. 1 mile time was around 05:00 minutes. Musculoskeletal: Improved flexibility, stiff muscles resolved, patient could do a split in 10 minutes. Mental symptoms: Insomnia resolved, mood swings resolved, difficulty thinking and understanding resolved. Neurological symptoms: Sensation of pins and needles resolved, light sensitivity resolved, mood swings 90% resolved. Gastrointestinal symptoms: Constipation resolved and quality of stool optimal, bloating resolved. Genitourinary symptoms: Urination was still slow, 90% improved. Vitals: High pulse rate resolved, low body temperature resolved.
July 2022 to present: Regrowth of good bacteria	The patient started to heal the mucosal lining and regrow good bacteria.
Symptoms	Constitutional: All of the patient’s symptoms are completely resolved. His body composition is still changing due to an increase in muscle mass. patient’s body weight is 185 pounds with a 29.5-inch waist. He has approximately normal blood laboratory tests, and he takes no medications. Musculoskeletal: Flexibility improved significantly, and can stretch into splits in about 3 to 5 minutes. As the patient timed in a 4.38-second 40-yard dash, he tore his Achilles heel. Patient’s heel shows significant signs of improved recovery with no surgery or physical therapy. Nutritional deficiency test on 12/2022 showed 90% absorption of nutrients.
Protocol used from January 2022 to present	Diets and supplements are varied and multifaceted.

Appendix E

Laboratory Test Results Before and After the Treatment

Appendix F

Appendix G

Appendix H

Appendix I

Appendix J

Appendix K

Appendix L

Appendix M

Appendix N

Patient Chart Notes

Appendix O

Appendix P
